# Validation of the Austin Assessment: A screening tool for cerebral visual impairment related visual issues

**DOI:** 10.1371/journal.pone.0293904

**Published:** 2023-11-02

**Authors:** Nicola McDowell, Philippa Butler

**Affiliations:** Institute of Education, Massey University, Auckland, New Zealand; LV Prasad Eye Institute, INDIA

## Abstract

Cerebral visual impairment is the most common cause of vision impairment affecting children in the economically developed world with a prevalence rate of approximately 3.4%. Currently there are limited options for screening for cerebral visual impairment, resulting in many children going undiagnosed, especially those that have normal visual acuity. The aim of this research was to validate an iPad App called the Austin Assessment, which was developed as a potential screening tool for cerebral visual impairment related visual issues. The research involved three separate phases: (1) creating a database of normative ranges for children aged 5–18 across the different variables of the Austin Assessment, (2) using the Austin Assessment to screen children aged 5–13 to assess the effectiveness of the Austin Assessment as a screening tool for CVI related visual issues, and (3) conducting specific validation research assessing children using the Austin Assessment and an already validated visual search tool. Each phase used different quantitative research methodologies to help show the effectiveness of the Austin Assessment as a screening tool for cerebral visual impairment related visual issues. From phase one of the research, thresholds were established for three variables of the Austin Assessment for the age groupings of 5–8, 9–12 and 13–18. If a child meets one of these thresholds this indicates further assessment is required to determine if they do in fact have cerebral visual impairment related visual issues. Phase two identified 17 children out of 270 who had clinical findings indicating visual issues; potentially indicative of CVI; investigation into the nature of these visual issues is ongoing. Phase three found that the Austin Assessment has moderate diagnostic value for each age group, with good sensitivity and specificity, making it effective at distinguishing those children who have visual issues from those who have typical vision. Further investigation is needed to confirm this initial validation.

## Introduction

Cerebral visual impairment (CVI) has been defined as a verifiable visual dysfunction which cannot be attributed to disorders of the anterior visual pathways or any potentially co-occurring ocular impairment [1]. CVI has long been recognized as the most common cause of visual impairment in children in the economically developed world [1]. More recently it has been recognized that CVI is also a threat to vision in developing nations as well [2, 3]. Visual issues reported to be part of CVI include reduced visual acuity (of non-ocular cause), visual field deficits, oculomotor disorders, abnormal crowding ratio (difference between visual acuity tested with single and crowded optotypes), impaired motion detection, and many visuo-cognitive or visuoperceptual impairments (higher visual functions) [4]. With the increased awareness in the CVI research and education communities of a prevalence rate of approximately 3.4% of children in mainstream education having CVI related visual issues [4], attention is now turning to effective methods for assessing and diagnosing CVI. However, as there is currently no international agreement on a CVI definition, different countries and even different organizations within countries have adopted their own definitions and diagnostic criteria for CVI [5]. This has caused some confusion for clinicians as to the best assessment protocol for diagnosing CVI and has resulted in many children with CVI related visual issues going undiagnosed.

To support awareness in this area, Boonstra, Bosch [6] have proposed a multidisciplinary approach to assessing and diagnosing CVI, which includes medical history and CVI questionnaires, ophthalmological and orthoptic assessment, neuropsychological assessment, neuroradiological evaluation and magnetic resonance imaging, and genetic assessment. In addition to these five areas, McConnell, Saunders [7] have also shown that other areas assessed in the diagnostic process include visual behaviors and direct observation, visual perception tests, ocular movement and posture assessments, IQ assessments, clinical electrophysiology, and neurodevelopmental tests. Further to this, Pilling, Allen [8] have recently outlined a checklist to help guide practitioners to decide whether CVI is a possibility that requires further CVI specific assessment. They outline that CVI may be present if two or three of the following criteria are met: 1) presence of risk factor(s), 2) reported or observed atypical visual behaviors, and 3) verifiable visual dysfunction on examination. The authors also outline that CVI is highly likely to be present if all three criteria are met [8].

An additional challenge in the identification and diagnosis of CVI is the cohort of children that have normal or near normal visual acuity but have significant issues with perceptual processing impairments relating to visual identification, visuospatial processing and attention, called higher visual functions (due to their higher order in the visual process). These higher visual functions are associated with the visual pathways mediated by the dorsal and ventral streams of visual processing [9]. The dorsal stream runs from the occipital lobe to the posterior parietal cortex at the top of the cerebral hemispheres [10, 11]. When there is bilateral injury to the posterior parietal lobes, it causes dorsal stream dysfunction. Common visual difficulties associated with a dorsal stream dysfunction include; simultanagnosia (an inability to see more than a few objects at a time), optic ataxia (impaired visual guidance of movement), and apraxia of gaze (the inability or difficulty with moving the eyes from one visual target to another) [12–14]. The ventral stream of visual processing, runs from the occipital lobe to the bottom and sides of the cerebral hemispheres in the inferior temporal region [10, 11]. When there is a bilateral injury in this area, the associated visual issues include; an inability to recognise text, objects and familiar people, difficulty with negotiating complex environments and difficulty with recognising faces and reading facial expressions [13, 15, 16].

Both a ventral and dorsal stream dysfunction result in visual perceptual deficits that greatly impact a child’s development and functioning [17, 18]. It has recently been shown that children with issues with the higher visual functions caused by a dorsal stream dysfunction have diminished visual parallel processing abilities, which greatly impacts their visual functioning and ability to engage with classroom activities [5, 19]. However, for these children to have the opportunity to be assessed and diagnosed with CVI (specifically issues with the higher visual functions or a dorsal stream dysfunction), they firstly must be recognized as having a visual issue. In New Zealand, as in other countries around the world, visual screening is conducted on all 4 year old children prior to starting school [20]. However, as the main focus of this screening is on visual acuity, children with higher visual function issues and typical visual acuity are unlikely to be identified through this process. In addition, issues with the higher visual functions or visual perceptual difficulties are not assessed in most pediatric eye clinics, so even if a child did undergo a more thorough vision assessment, it is unlikely that their CVI related visual issues would be identified [21]. This gives rise to the need for a simple and effective screening process that can be used to help identify children who potentially have CVI related visual issues that warrant further assessment.

One method of screening that has been proposed is structured history taking, specifically the Five Questions [22] that were derived from the 50 item CVI inventory [23] and the CVI Questionnaire [24]. The aim of the history taking approach is to solicit vital information about the child’s behavior and functioning in different environments based on parental observations [25]. Both the Five Questions and the CVI questionnaire have been shown to be effective screening tools for CVI related visual issues and have the potential to be used for population screening for CVI [26]. More recently, the 50-item inventory has been expanded to include more questions (a total of 56) and renamed the Higher Visual Function Question Inventory (HVFQI). Within the HVFQI, a subset of 11 questions have been identified as a quick screening process, as they very quicky discriminate between children with CVI related visual issues versus those with typical vision [18]. However, as this approach of CVI questioning does not include a direct observation of a child’s visual functioning, it only provides subjective evidence of visual issues [27]. Another option for CVI screening could therefore be a simple and easy-to-conduct effective assessment tool that allows for observation or analysis of a child’s visual functioning to provide objective evidence, which can support the subjective parent responses from the screening questionnaires [27].

With this in mind, a screening tool to detect CVI related visual issues, specifically, limitations in the higher visual functions of catering for visual crowding and shape analysis was developed, called the Austin Assessment [28]. The Austin Assessment is a simple iPad activity of matching cards with different shapes over 5 levels, with the numbers of cards increasing from 4 cards and 1 pair at level one, to 12 cards and 5 pairs at level five [28]. The activity is completed via an app on an iPad and requires the child to use their finger to move a card on top of another card when they believe they are a pair. If a child moves two non-matching cards together, they still appear to have matched, so there is never a sense of failure. There is a multi-colored version of the app, with the cards being a mixture of blue, red, green and yellow shapes (one color per card) and a single-colored version, where all the shapes are the same color (either blue, red, green or yellow). These two options allow for the assessment of children with a range of abilities (some children find the color helps with matching, while others find it distracting). The Austin Assessment App guides participants through the assessment process and does not require external instruction from a person with specific vision knowledge. The Austin Assessment App was designed to capture key indicators of issues with the higher visual functions or visual perceptual difficulties, including darting eye movements, slower processing of the visual scene with increasing complexity [5, 19, 29], a trend of worsening performance with increased task demand, impaired search performance [5, 17], slower responses to visual stimuli [5], difficulties with visually guided movement [10, 19], and behavioral responses such as being easily distracted by competing sensory stimuli [30] and increasing levels of frustration and/or anxiety [31].

The Austin Assessment App measures the impact these key indicators have on a child’s performance through three variables. These include overall time taken to complete all five levels, accuracy in matching pairs at each level, and the time taken to match the first pair at each level (dwell time). Due to the heterogenous nature of CVI, in that it can manifest and present differently in each individual child [32, 33], the measurement of these three variables helps to ensure that all children that warrant further assessment to determine if they do have CVI related visual issues are identified. Some children can be faster at completing the assessment, but less accurate, while others can be slower and more accurate. Moreover, some children can have a slow response time when first seeing the cards, while others do not. A fourth variable, eye movement, is also being tracked but an accurate way to quantitively analyze this is still being developed (to access the eye tracking function of the app, the device needs to have Apple’s True Depth Camera). Initial research on the Austin Assessment showed that children with CVI related visual issues took twice as long to match the pairs at each level, were less accurate, took longer to match the first pair at each level, and had increased darting eye movements [28]. This research also showed the Austin Assessment to be an effective assessment of visual performance that differentiated between children with CVI related visual issues and those with typical vision [28]. As the Austin Assessment App does not need to be conducted by a person with specific vision expertise, it can be facilitated by individuals, parents, teachers, therapists, and other professionals. The purpose of the research reported in this article was to therefore provide an initial validation of the Austin Assessment as an effective screening tool for CVI related visual issues in children.

## Methods

The validation process for the Austin Assessment involved three distinct research phases using quantitative research methodologies, including:

1. creating a database of normative ranges for children aged 5–18 across the different variables of the Austin Assessment;
2. using the Austin Assessment to screen children aged 5–13 to assess the effectiveness of the Austin Assessment as a screening tool for CVI related visual issues; and
3. conducting specific validation research assessing children using the Austin Assessment and an already validated visual search tool.

All three phases were approved separately by a University Human Ethics Committee. Written parental and child consent and verbal child assent was obtained for all participants for all three phases.

### Phase one

Currently, the Austin Assessment App can generate data for three of the four variables of interest: overall time to complete all five levels, accuracy in completing each level, and time taken to match the first pair at each level (dwell time). Eye movement while completing each level is recorded as a diagram that can be assessed qualitatively, but it is not yet available as quantitative data. To determine the thresholds where further visual assessment is required for the three variables, 724 children were assessed using the AA App (Table 1). The children were aged between 5–18 and were from four different schools in New Zealand. At the secondary school (children in years 9 to 13, aged 13–18), two form classes were randomly picked for each year level and parents were invited to consent to their child participating in the research. For the intermediate school (children in years 7 and 8, aged 11–13), two classes for each year group were randomly picked and parents were invited to consent to their child participating. For the two primary schools (one catering for children in years 1 to 8, aged 5–13, and the other for children in years 1 to 6, aged 5–11), the entire school was invited to participate in the research.

**Table 1:** Age and gender of normative range database participants.

	Female		Male		Prefer not to say		Total	
	n	%	n	%	n	%	n	%
5 years	30	9.0%	28	7.3%	0	0.0%	58	8.0%
6 years	39	11.7%	58	15.1%	0	0.0%	97	13.4%
7 years	29	8.7%	38	9.9%	0	0.0%	67	9.3%
8 years	26	7.8%	44	11.4%	0	0.0%	70	9.7%
9 years	31	9.3%	39	10.1%	0	0.0%	70	9.7%
10 years	24	7.2%	26	6.8%	0	0.0%	50	6.9%
11 years	44	13.2%	32	8.3%	1	20.0%	77	10.6%
12 years	29	8.7%	43	11.2%	0	0.0%	72	9.9%
13 years	15	4.5%	26	6.8%	2	40.0%	43	5.9%
14 years	21	6.3%	14	3.6%	0	0.0%	35	4.8%
15 years	13	3.9%	15	3.9%	0	0.0%	28	3.9%
16 years	14	4.2%	9	2.3%	1	20.0%	24	3.3%
17 years	17	5.1%	9	2.3%	1	20.0%	27	3.7%
18 years	2	0.6%	4	1.0%	0	0.0%	6	0.8%
**Total**	**334**	**100.0%**	**385**	**100.0%**	**5**	**100.0%**	**724**	**100.0%**

**Fig 1:**
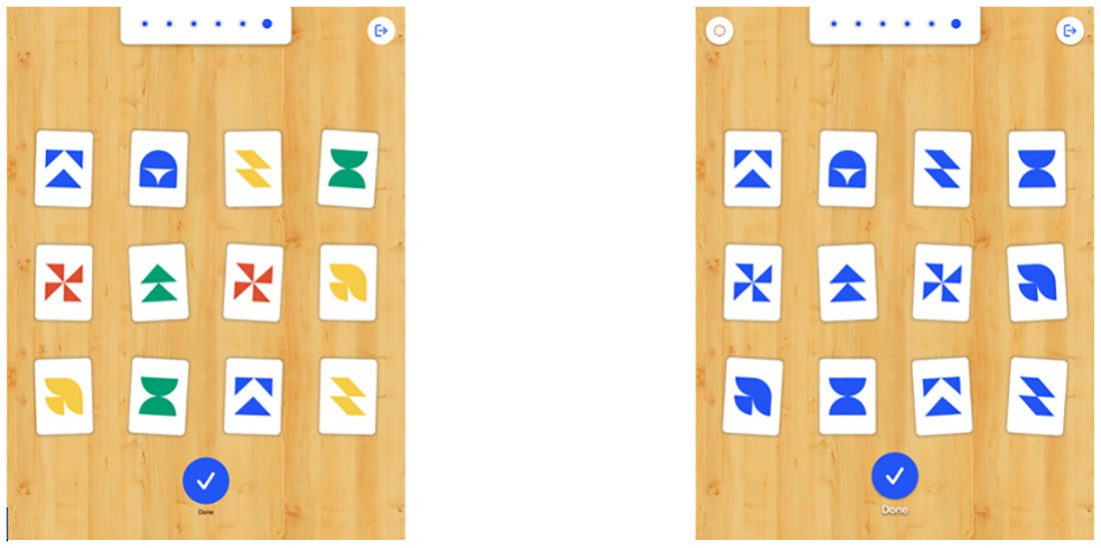
Austin Assessment multi and single colored versions.

Each child completed the Austin Assessment twice, once using the multi-colored version and once using the single-colored version in blue (Fig 1). The App uses images and icons showing the children what to do and they complete a tutorial level before starting on the actual assessment. Minimal verbal instruction is required. Once a child had completed each assessment, their date of birth, gender and any known relevant conditions were entered into the App, along with a research code to ensure anonymity. All assessments were conducted by trained research assistants and the lead researcher.

The normative range data were analyzed using a simple linear regression to help determine age groups. Percentiles for the overall time and dwell time measures were calculated to identify thresholds considered indicative of a potential visual search issue for each age group.

### Phase two

The Austin Assessment App was used to screen 271 children aged between 5–13 in one school (the results were also included in the normative range database). Parents of children attending the school were invited to consent to their child participating in this research. Just over half of the children at the school (55%) participated and each child was assessed twice, once with the multi-colored version and once with the single-colored version. As with the normative range data collection, each child’s date of birth, gender and any known relevant conditions were entered into the app, along with a research code to ensure anonymity. All assessments were conducted by trained research assistants and the lead researcher.

Following the assessments, the thresholds developed in the normative range database (phase one) were used to identify 26 children who warranted further assessment to determine if they did have CVI related visual issues. There are a total of six individual thresholds from the two versions of the Austin Assessment (multi-colored and single-colored) and the three variables (overall time taken, accuracy and dwell time). Only one of these variable thresholds needs to be met for a child’s performance to be flagged as needing further assessment. This is due to the heterogeneity of CVI and the need to allow for a wide range of presentation of the visual issues [32, 33]. Twenty-three of these children underwent further visual assessment at the school. During this process, each child was seen by an ophthalmologist, an orthoptist, and the lead researcher, who is an experienced vision education and O&M specialist, who each carried out a range of tests to develop an overall picture of the child’s visual functioning (Table 2). Further review of the child’s medical records was also carried out by the ophthalmologist when appropriate after gaining consent from the parents. Results from the testing were collated and analyzed by the ophthalmologist and the lead researcher.

**Table 2:** Vision assessments.

Ophthalmologist	Orthoptist	Teacher of pupils with Visual Impairment / Orientation and Mobility Specialist
RAPD (Relative afferent pupillary defect)	Distance VA Threshold test R & L 6/9 HOTV* *(6/9 (measured in meters) threshold is the equivalent of 20/30 (feet) or 0.18 LogMar)	Hiding Heidi (Low contrast face test)
Ocular motility Media opacity	+2 fogging 6/24 threshold* *(6/24 (measured in meters) threshold is the equivalent of 20/80 (feet) or 0.60 LogMar	Higher Visual Functions Question Inventory 11 Questions
Optic disc examination	Near acuity (binoc) 6/12 threshold* *(6/12 (measured in meters) threshold is the equivalent of 20/40 (feet) or 0.30 LogMar)	Beery visual perceptual test
Visual fields: FDT C20 screening each eye Medical history, review of hospital record	Colour each eye: Ishihara 2 plates incorrect Autorefraction screening using the Plusoptix, with Retinomax	Cancellation test (Teddy bear cancellation 5–8, single letter cancellation for 9–13)

### Phase three

The aim of phase three was to use specific validation methodology to assess children with CVI related visual issues to help validate the Austin Assessment. However, due to the challenge of many children in New Zealand with CVI related visual issues going undiagnosed, especially if they have normal or near normal visual acuity (as with many other countries) [26, 28, 34], it was recognized that it would be difficult to identify a large population of children already diagnosed with CVI who had good enough visual acuity and the cognitive ability to complete the Austin Assessment. With the awareness that CVI related visual issues have been identified in children with conditions such as being born premature [35], neurodevelopmental disorders [36], cerebral palsy [37], and genetic or chromosomal conditions [38, 39], it was determined that the validation research could be conducted on a population of children with these conditions. The validation research process involved assessing children with two assessments, the Austin Assessment and an already validated visual search test, and comparing the performance between the two tests, using the performance in the validated test to validate the Austin Assessment. After careful consideration, a cancellation test was identified as being the most similar to the Austin Assessment and chosen as the visual search assessment, as it requires participants to search for a specific item surrounded by a number of distractors [19, 40, 41]. The cancellations tests with normative data for children aged 5–16 included the Teddy Bear Cancellation Test (TBCT) (children aged 5–8) [40] and the Six Letter Cancellation Task (SLCT) (children aged 9–16) [41].

Using a statistical calculation, it was determined that at least 134 children would need to be assessed for this method to be effective. Out of those 134 children, approximately 30 needed to have typical vision with the rest of the sample size having a range of visual abilities. To ensure this range, the research invitation asked for participants who had CVI related visual issues, were born premature, or had conditions including neurodevelopmental disorders and cerebral palsy. For the children with typical vision, 28 children were randomly selected to complete both the cancellation test and the Austin Assessment in the primary school where the screening research (phase two) was conducted. To select these children, every 6^th^ name on the class lists was highlighted and the lead researcher conducted both tests on these children at the same time as the other children were being assessed with just the Austin Assessment. To reach children aged between 13–18 with typical vision, siblings of the participants with conditions were invited to participate to ensure the participants with typical vision covered the full age range of 5–18.

The 23 children identified for further assessment in the screening research were also included in the validation research and the lead researcher conducted the cancellation tests with them during the vision assessment process. To obtain the other participants, the research was advertised in relevant New Zealand Facebook groups, including Parents of Visually Impaired (PVI), Very Important Parents (VIP), Autism NZ, and Home Education in NZ. The lead researcher travelled around New Zealand to assess all children in the validation research.

**Fig 2:**
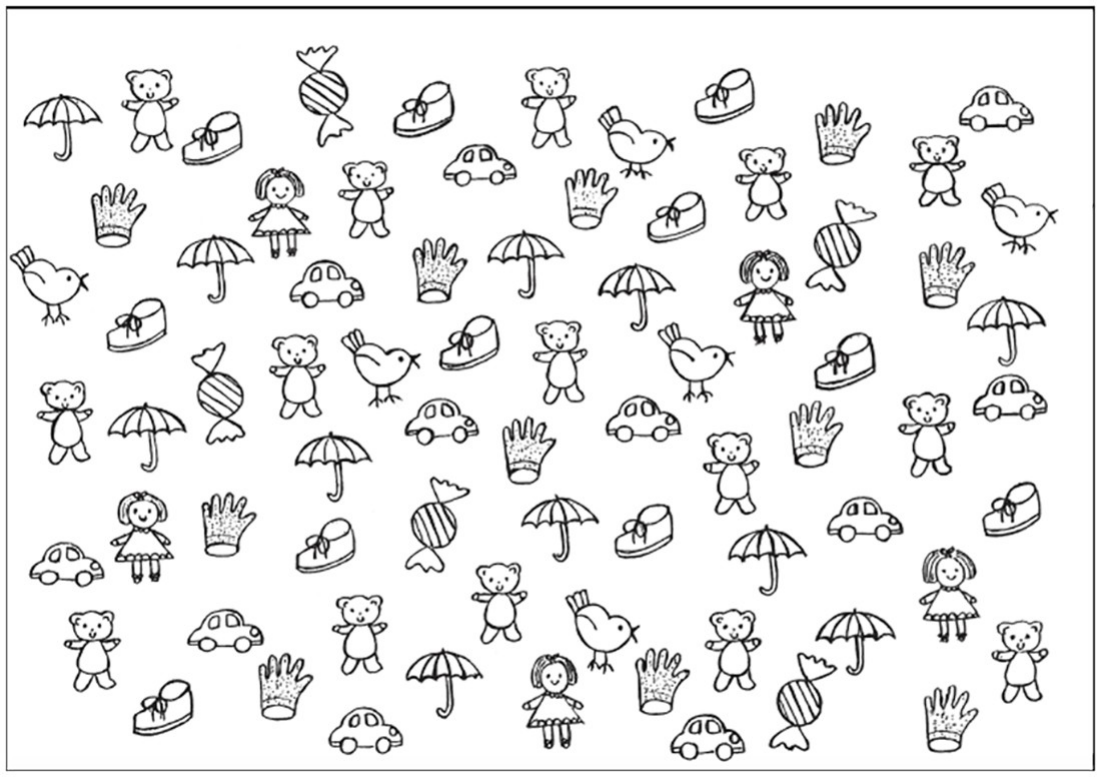
Teddy bear cancelation test.

The process for conducting the assessments was the same for each child. Firstly, they completed the cancellation test appropriate to their age. For the TBCT, an A4 sheet was placed in front of the child (Fig 2). The lead researcher crossed out one teddy bear in the top left corner to model what the child needed to do. The child was then asked to locate all the teddy bears and cross them out or circle them. If the child stopped before all teddy bears were crossed out, they were asked once if they were finished. The task was completed when the child said they had finished [40]. On the TBCT there are a total of 15 teddy bears and 60 distractors distributed proportionally on the page in a pseudo-random array in five columns [40]. The columns are numbered 1–5 from left to right. The three variables used for the TBCT include the number of omissions (O), the location of omissions (LO-S), and the location of the first three teddy bears crossed out (START-S). Each column has a nominal value, -1 for columns 1 and 2, 0 for column 3, and +1 for columns 4 and 5 [40]. Each participant’s results were compared to the normative range for the TBCT for their age [40].

**Fig 3:**
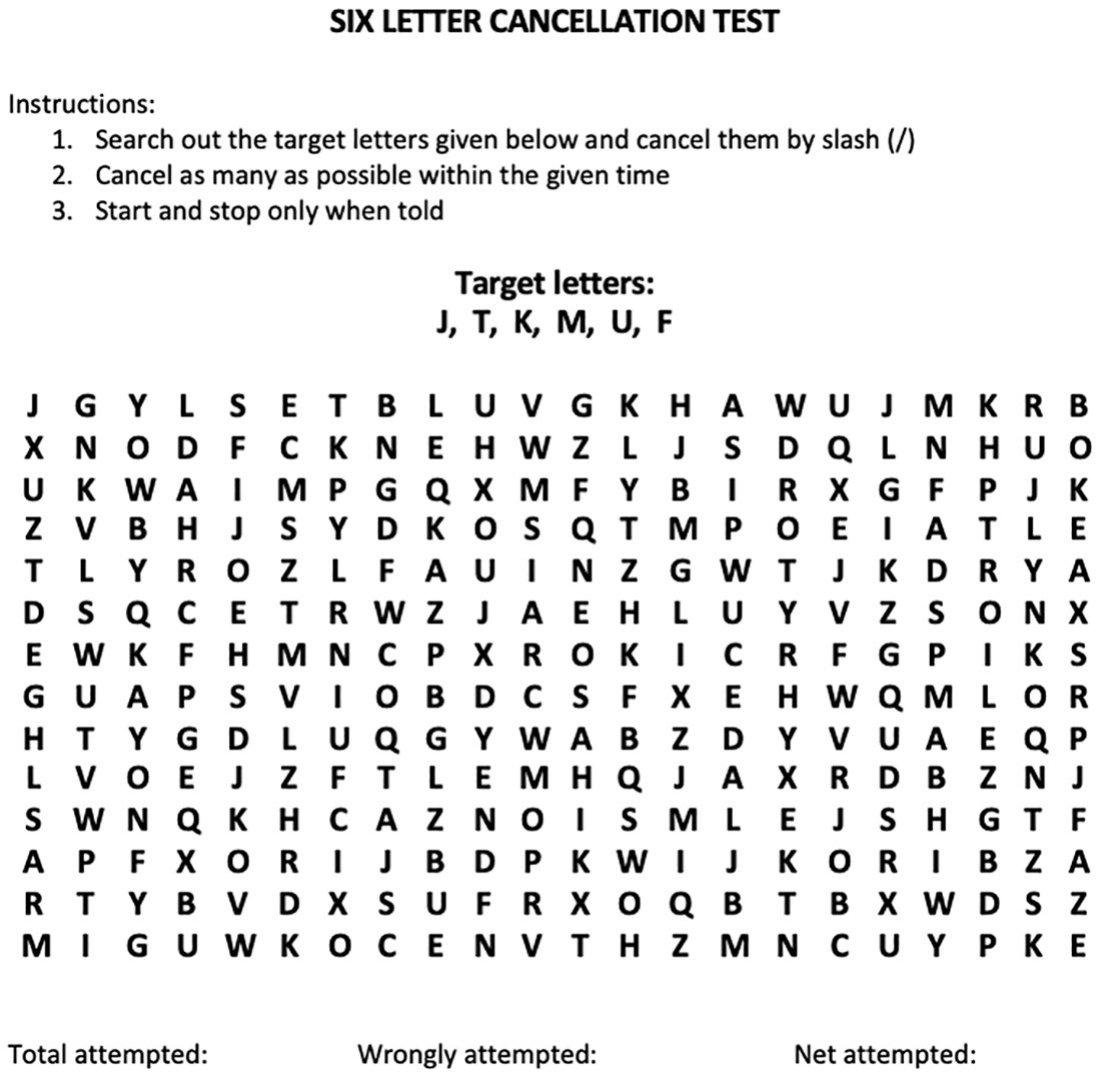
Six letter cancelation test.

For the SLCT, an A4 sheet of paper was placed in front of the child (Fig 3). The lead researcher pointed out the six target letters at the top of the page and asked the child to cancel as many of these as they could in 90 seconds in the working grid, which consisted of letters of the alphabet arranged randomly in 22 rows and 14 columns [41]. Each child was told they could use one of two methods, either look for one letter at a time or all six letters at the same time. They were also told they could follow a horizontal, vertical or a random path to search for the letters [41]. Each test was scored by calculating the total number of cancellations and subtracting the wrong cancellations for a net score. These scores were then compared to the normative range for their age [41]. Each child also completed the Austin Assessment twice, once using the multi-colored version and once using the single-colored version in blue.

The results of the cancellation tests were analyzed according to the protocols for the respective tests. For the TBCT, a result was considered outside the normative range if there were one or more omissions [40]. For the SLCT, a result was considered outside the normative range if it was at the 5^th^ percentile or less [41]. The individuals who had a result outside the normative range on the comparison tests were then compared to those who were outside the normative range of the Austin Assessment using a Cohen’s kappa test of agreement [42, 43].

The sensitivity and specificity [44, 45] for the Austin Assessment were also calculated via a kappa test for the participants in phase three, as the incidence of existing conditions related to visual search issues was known.

## Results

### Phase one

**Fig 4:**
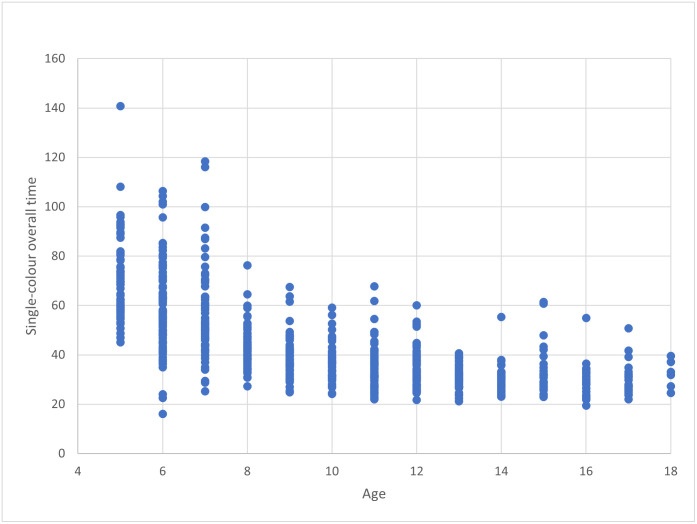
Single color scatterplot.

**Fig 5:**
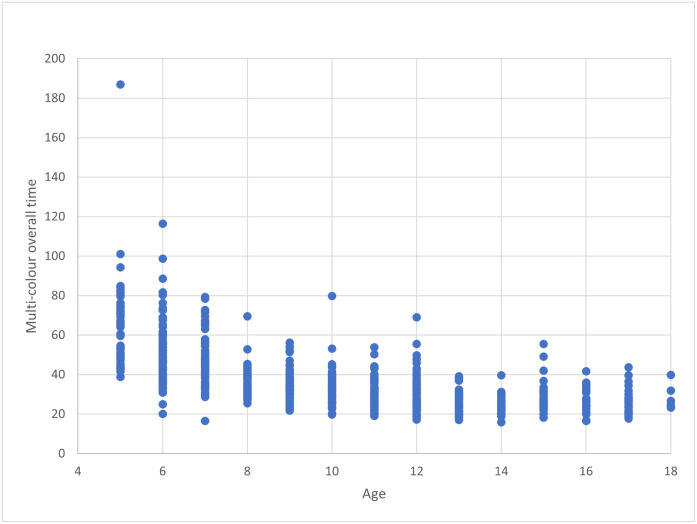
Multi color scatterplot.

A simple regression analysis was performed to determine the impact of age on overall time for each version of the Austin Assessment. For both versions, age (in years) was a significant predictor of overall time taken to perform the test (for the single-colored version, F(1,722) = 555.071, p < .001, R^2^ = .435; for the multi-colored version, F(1,722) = 441.340, p < .001, R^2^ = .379). For the single-colored version, overall time reduced by 3.34 seconds for each year of age, while for the multi-colored version, overall time reduced by 2.83 seconds for each year of age. Scatterplot diagrams showed three distinct groupings: children aged 5 to 8 years, aged 9 to 12 years, and aged 13 to 18 years (Figs 4 and 5).

For the overall time variable, the range in time taken to complete the five assessment rounds was greater for the 5–8 year old group (single-colored: 16–141 seconds; multi-colored: 16–187 seconds) than the 9–12 year old group (single-colored: 21–68 seconds; multi-colored: 17–80 seconds) and the 13 to 18 year old group (single-colored: 19–62 seconds; multi-colored: 15–56 seconds). For the dwell time variable, the average time ranged from 0.75 to 5.53 seconds (single-color) and 0.95 to 7.67 seconds (multi-color) for the 5–8 year old group, from 0.76 to 3.96 seconds (single-color) and 0.83 to 3.80 seconds (multi-color) for the 9–12 year old group, and from 1.37 to 1.94 seconds (single-color) and 1.16 to 1.93 seconds (multi-color) for the 13–18 year old group. For the accuracy variable, results ranged from 0 accurate rounds to 5 accurate rounds for each version of the test for each age group.

To determine the normative range thresholds, percentiles were calculated for the overall time and average dwell time variables. As the 5–8 year old group exhibited a greater range of times, the threshold was set at the 85^th^ percentile. For the 9–12 year old and 13–18 year old groups, the threshold was set at the 95^th^ percentile. These thresholds for the overall time and dwell time variables will be built into the Austin Assessment App, a proprietary piece of software. As such, they are not being reported in full here. For the accuracy variable, the threshold was set at less than 4 accurate rounds, for each age group. An individual was considered to warrant further visual assessment if they were outside the normative range on one or more of the variables, for either the single-colored version or the multi-colored version of the Austin Assessment.

### Phase two

The analysis of the vision assessments on the 23 children who warranted further visual testing identified that 17 out of 23 had a verifiable reason or clinical findings that would make a result that was outside the normative range in the Austin Assessment a reliable true positive, and not just for CVI related visual issues (Table 3). Using the criteria outlined by Pilling, Allen [8], five children were identified as CVI being highly likely (meeting all three criteria), and five children were identified as maybe having CVI (meeting two criteria). A further seven children were identified as having a visual issue, but further assessment was required to determine if this was ocular or brain based. Fifteen of these 17 children require further assessment with local eye specialists (optometrist and/or ophthalmologist) to confirm the nature of their visual issues. The remaining six children were false positives from the Austin Assessment.

**Table 3:** Phase two screening research vision assessment findings.

Child	Age	VA	VF	Color	HH	11?s	Beery	AA	Ophthalmic Findings / Follow up	CVI checklist
1	12	A	U	A	A	30	N	95^th^ percentile for time	Under care already, possible neurological visual issue, risk factor	3/3 CVI highly likely
2	6	N	U	A	A	26	A	85^th^ percentile for time & accurate 2 levels	Refractive error, astigmatism, optometrist referral	2/3 CVI possible
3	5	N	A	A	N	26	A	95^th^ percentile for time	Poor convergence, risk factor	2/3 CVI possible
4	10	N	N	A	N	24	N	95^th^ percentile for time	Refractive error, Strabismus, anisometropia, under care already, risk factor	3/3 CVI highly likely
5	8	N	A	A	N	21	N	95^th^ percentile for time	Possible right temporal hemianopia, left reduced sensitivity, ophthal referral	2/3 CVI possible
6	7	A	A	A	N	21	N	Accurate 1 level	Abnormal left optic disc, VF abnormal both eyes, risk factor, ophthal referral	2/3 CVI possible
7	7	N	A	N	N	15	A	Accurate 1 level	Refractive error, inferior VF loss both eyes, optometrist referral	2/3 CVI possible
8	7	N	A	N	N	8	N	Accurate 1 level	Right RAPD, mild refractive error, ophthal referral	1/3 CVI unlikely
9	8	N	A	N	N	6	N	Accurate 2 levels	VF abnormal right, random triggering left, refractive error, risk factor, optometrist referral	3/3 CVI highly likely
10	5	N	U	N	N	5	A	85^th^ percentile for time	Right disc and VF abnormal, ophthal referral	1/3 CVI unlikely
11	5	N	A	N	N	4	N	85^th^ percentile for time	Left optic disc abnormal with corresponding left VF abnormality, ophthal referral	1/3 CVI unlikely
12	10	N	N	N	N	17	N	95^th^ percentile for time	Both discs abnormal, left cupped, possible left Threshold VF inferior defect but isolated zone, risk factor, ophthal referral	3/3 CVI highly likely
13	6	N	A	N	N	12	N	85^th^ percentile for time	Refractive error, right disc and VF abnormal, could be both VF (bitemporal). Gest 37/40, ophthal referral	1/3 CVI unlikely
14	5	N	N	N	N	10	N	85^th^ percentile for time	Refractive, VF (possible bitemporal), optom referral	1/3 CVI unlikely
15	12	A	N	N	N	8	N	Accurate 2 levels	Refractive error, optometrist referral	1/3 CVI unlikely
16	7	N	A	N	N	2	N	Accurate 2 levels	VF could be abnormal, optometrist referral	1/3 CVI unlikely
17	6	A	A	A	A	NA	NA	Accurate 0 levels	Global developmental delay, possible disc pallor, optometrist referral	3/3 CVI highly likely
18	7	N	N	N	N	15	N	85^th^ percentile for time	Nothing to note	
19	8	N	N	N	N	12	N	95^th^ percentile for time	Nothing to note	
20	8	N	N	N	N	11	N	95^th^ percentile for time	Nothing to note	
21	5	N	N	N	N	8	N	85^th^ percentile for time	Nothing to note	
22	7	N	N	N	N	7	N	85^th^ percentile for time	Nothing to note	
23	6	N	U	N	N	2	N	95^th^ percentile for time	Nothing to note	

Headings key: VA: Visual Acuity, VF: Visual Field, HH: Hiding Heidi, 11?’s: Higher Visual Function Question Inventory 11 screening questions, Beery: Developmental test of visual motor integration, AA: Austin Assessment, CVI checklist [8].

(Table key: A = abnormal, N = normal, U = unreliable)

### Phase three

For the validation research, a total of 149 children aged between 5–18 were assessed using the Austin Assessment and the age-appropriate cancellation test (either TBCT or SLCT). The range of conditions that the children had included neurodiversity (autism, ADHD, auditory processing disorder, dyslexia, dyspraxia, dysgraphia, dyscalculia), premature infants, cerebral palsy, Down’s syndrome and CVI (Table 4).

**Table 4:** Validation research participants.

	Frequency	Percent of total conditions	Percent of 110 participants with identified conditions
Visual issue identified through Austin Assessment screening research	17	13.2%	15.5%
CVI	13	10.1%	11.8%
Neurodiverse	70	54.3%	63.6%
Cerebral palsy	6	4.7%	5.5%
Born premature, before 32 weeks	19	14.7%	17.3%
Other conditions	4	3.1%	3.6%
Total	129	100.0%	117.3%

Notes: An individual child could present with more than one condition.

Neurodiverse includes ADHD, autism, auditory processing disorder, sensory processing disorder, dyslexia, dyspraxia, dysgraphia, dyscalculia.

Other conditions include Down’s syndrome, global developmental delay, and intellectual disability.

39 participants did not have an identified condition, including 6 participants identified in the Austin Assessment screening research where further expert screening excluded a visual issue.

Kappa tests of agreement were used to compare the number of individuals in each age group who were identified by the Austin Assessment as having a potential visual issue, with the number of individuals who were outside the normative ranges for the TBCT or the SLCT (Table 5). For the 5–8 age group, there was slight (but not significant) agreement between the Austin Assessment and the TBCT (κ = 0.091, n = 53, p = 0.347), with a sensitivity of 0.727 (95% CI [0.464, 0.990]) and a specificity of 0.429 (95% CI [0.279, 0.578]). For the 9–12 age group, there was moderate agreement between the Austin Assessment and the SLCT (κ = 0.512, n = 63, p<0.001), with a sensitivity of 1.0 (95% CI [1.0, 1.0]) and a specificity of 0.917 (95% CI [0.847, 0.987]). For the 13–18 age group, there was slight agreement between the Austin Assessment and the SLCT (κ = 0.343, n = 33, p = 0.032), with a sensitivity of 0.750 (95% CI [0.450, 1.050]) and a specificity of 0.680 (95% CI [0.497, 0.863]).

**Table 5:** Comparing AA and cancelation tests.

		AA: Visual issue identified	AA: No issue identified	Total
5–8 age group	TBCT: Visual issue identified	8	3	11
	TBCT: No issue identified	24	18	42
	Total	32	21	53
9–12 age group	SLCT: Visual issue identified	3	0	3
	SLCT: No issue identified	5	55	60
	Total	8	55	63
13–18 age group	SLCT: Visual issue identified	6	2	8
	SLCT: No issue identified	8	17	25
	Total	14	19	33

The diagnostic value of the Austin Assessment was examined for the participants in phase three, as the group included individuals with conditions that are known to have visual issues as well as individuals with no known conditions (Table 6). The information about the 63 neurodiverse participants who had no other pre-existing condition was inconclusive (47 individuals were not identified with a visual issue by either the Austin Assessment or the comparative test). The case could not be made that neurodiversity equated with a pre-existing visual issue, so these individuals were excluded from this analysis. For the 5–8 age group, the Austin Assessment had moderate diagnostic value (κ = 0.571, n = 36, p < .001), with a sensitivity of 0.857 (95% CI [0.707, 1.007]) and a specificity of 0.462 (95% CI [0.191, 0.733]). For the 9–12 age group, the Austin Assessment had moderate diagnostic value (κ = 0.475, n = 16, p = 0.049), with a sensitivity of 0.533 (95% CI [0.281, 0.786]) and a specificity of 1.0 (95% CI [1.0, 1.0]). For the 13–18 age group, Austin Assessment had fair diagnostic value (κ = 0.339, n = 34, p = 0.041), with a sensitivity of 0.727 (95% CI [0.464, 0.990]) and a specificity of 0.800 (95% CI [0.449, 1.151]). The number of participants in each age group is too small to reach a conclusive judgment of the diagnostic value of the Austin Assessment, but overall, the results indicate that the assessment shows good sensitivity and specificity. This means that for each age group the Austin Assessment is likely to accurately identify that an individual does have a visual issue (sensitivity = 85.7% for the 5–8 age group, 53.3% for the 9.12 age group, 72.7% for the 13–18 age group), and accurately identify that an individual does not have a visual issue (specificity = 46.2% for the 5–8 age group, 100% for the 9–12 age group, 80% for the 13–18 age group). More investigation is warranted.

**Table 6:** Diagnostic value of the Austin Assessment.

		Condition present	No condition	Total
5–8 age group	AA: Visual issue identified	18	7	25
	AA: No issue identified	3	6	9
	Total	21	13	34
9–12 age group	AA: Visual issue identified	8	0	8
	AA: No issue identified	7	21	28
	Total	15	21	36
13–18 age group	AA: Visual issue identified	8	1	9
	AA: No issue identified	3	4	7
	Total	11	5	16

## Discussion

The three-phase approach to the validation of the Austin Assessment has allowed for a thorough evaluation of the App as a screening tool for CVI related visual issues for children aged from 5–18. This approach has also allowed for the constant refinement and ongoing development to improve the functionality of the App in a range of different settings, which has helped to prepare the App for future use around the world. However, while each phase of the research process had a specific purpose in terms of validating the Austin Assessment App, the research also encountered several issues relating to the assessment and diagnosis of CVI, highlighting that screening of this complex condition is only one aspect of the current CVI landscape that needs to be addressed. However, accurate screening for CVI related visual issues will help to identify the true prevalence of this condition around the world and help to better understand the nature and impact of the different visual issues experienced by those with CVI.

The large numbers of children assessed for the normative range database in phase one of the research and the regression analysis conducted on the data for each age group helped to provide clear thresholds for when further assessment is required for the overall time taken to complete the assessment, the accuracy in completing the assessment, and the dwell time (time taken to match the first pair at each level). The finding that children became faster at both the multi-colored and single-colored version of the Austin Assessment with each year of age was an expected outcome as this mirrors the development of visual attention in childhood [46–48] and is in line with the observation of the research assistants who conducted the assessments of the children with a more chaotic approach to completing the Austin Assessment in the younger age groups. The natural groupings of the 5–8 year olds, 9–12 year olds, and 13–18 year olds is also in line with the expected maturation of visual attention abilities during childhood [47, 48].

Although the thresholds for further assessment were established through robust statistical analysis of the phase one data, it was also important to test each threshold in a real-world context. Phase two of the research allowed for this and also provided an opportunity for a small-scale assessment of the effectiveness of the Austin Assessment App as a screening tool for CVI related visual issues. Unfortunately, due to the COVID pandemic, the numbers of participants in phase two was smaller than anticipated, however the data gathered from this phase has still provided important and useful information on the effectiveness of the Austin Assessment. Another challenge encountered in phase two of the research related to the assessment and diagnostic process for children with CVI related visual issues. In New Zealand, as with many countries around the world, children with normal visual acuity and higher visual function issues are not often being referred to pediatric eye specialists to assess for CVI related visual issues. Although Simkin and Ziaei [49] recommend a low threshold in a general practitioner (GP) clinical setting for referral of pediatric ocular conditions for further detailed examination, the basic visual tests carried out by GPs (visual acuity, red reflex test, ophthalmoscope examination) [49] may not identify issues with the higher visual functions and therefore not refer children with normal visual acuity and higher visual function issues for further assessment.

The children that do get referred for an ophthalmic assessment for suspicion of CVI are generally those with low visual acuity and a known medical history that is suggestive of CVI. However, as highlighted by Pilling, Allen [8] while a known medical risk factor can be an indicator for CVI, not all children with CVI will present with a known medical event that resulted in their visual issues. This is supported by Chandna, Ghahghaei [18] who highlight that normal visual acuity and absence or presence of neuroimaging findings no longer excludes a diagnosis of CVI. In their research, 23% of the children with CVI related visual issues had normal MRIs [18]. However, in New Zealand, as with many countries, a CVI diagnosis is still reliant on proof of brain injury through neuroimaging (MRI) [50], but to be referred for a publicly funded MRI in an overburdened medical system, there needs to be a known medical risk factor for CVI (i.e. premature birth, brain injury). This creates an environment where it is challenging for a child with normal visual acuity, but significant issues with their higher visual functions, to even be assessed for CVI, let alone receive a diagnosis of CVI. It also means that clinicians may be unsure of how to assess a child with normal visual acuity and higher visual function issues if they do present at an ophthalmology clinic.

To try and overcome this, the main researcher worked with a New Zealand based pediatric neuro ophthalmologist to develop an appropriate protocol to assess the children that met a threshold for further assessment in phase two of the research, within the constraints of not being in a clinical setting (the research was conducted in a different town to where the ophthalmologist was based) and not having the funding for extensive medical examinations such as MRI. As suggested by Boonstra, Bosch [6] and McConnell, Saunders [7], the protocol included multi-disciplinary assessments of medical history and CVI questionnaires (due to time constraints only the 11 questions from the HVFQI were used), ophthalmological and orthoptic assessment, visual perception tests (Beery) and ocular movement assessment. The Beery Visual Motor Integration Test was recommended for the visual perception test due to its potential for helping to identify issues with the higher visual functions, specifically a dorsal visual stream dysfunction. Although this protocol did not include all the areas outlined by Boonstra, Bosch [6] and McConnell, Saunders [7], referrals were recommended for when further assessment was needed if possible within the New Zealand medical system.

The analysis of the findings from the 23 children who underwent the assessment protocol provided some interesting findings that reflect the complex nature of CVI. While there were some similarities and expected findings (4 out of the 23 had abnormal visual acuities, 14 out of the 23 had either unreliable or abnormal visual fields), there were also unexpected findings (3 out of the 23 had contrast sensitivity issues, 7 out of 23 had color issues, 4 out of 23 had an abnormal result in the Beery test). The abnormal color results is an interesting finding, as although it has been recognized that children with CVI related visual issues may have color issues [51], it is not often reported. The low number of children having an abnormal result in the Beery test highlights the issue that although this can be a useful test to use, the actual purpose of the test is to assess the extent to which individuals can integrate their visual and motor abilities, not to assess visual perception [52]. A more focused visual perceptual test should therefore be considered. Overall, each child’s assessment picture was also completely different, highlighting the heterogenous nature of CVI [18] and supporting the idea that each child needs to have an individual CVI profile created outlining their specific visual issues [9]. However, due to the fact that only four children had abnormal visual acuity, most of these children would not have even been considered for further vision testing following the visual screening undertaken prior to them beginning school unless their parents had been concerned about their child’s visual behaviors and requested follow up assessments.

The scores for the 11 questions from the HVFQI for each child also provides useful information in relation to the potential benefits of combining the 11 questions and the Austin Assessment App for screening for CVI related visual issues. Eight out of the 10 children who scored either a 2 or a 3 on the CVI checklist (meaning that CVI is possible/highly likely) [8], also had a total score of over 15 for the 11 questions. Although Chandna, Ghahghaei [18] have not provided a total score on the 11 questions that indicates CVI is highly likely, a score of over 15 suggests a number of responses to the 11 questions have either been ‘always’ or ‘often’, indicating an issue with higher visual functions [18]. For the remaining two children, one child was supported by his teacher aide (who had only just started working with him) at the assessment and she was unable to answer the questions, and the other child’s parents had not suspected their child had a visual issue prior to this research. However, in the follow up discussion where the findings from the vision assessment was shared with them, they were able to report noticing issues with their child’s lower visual field that they had not considered when answering the relevant questions in the 11 questions screening. All of these 10 children also met the threshold for further assessment in either time taken or accuracy at the higher end of the scale (well over the 95% percentile or accurate in only 1 or 2 levels), meaning that their performance on the Austin Assessment was furthest from the norm. This highlights that the Austin Assessment has the potential to provide subjective evidence of visual issues to support the objective evidence provided by parents in the 11 questions, creating an effective combined option for screening for CVI related visual issues.

An unexpected finding from phase two of the research, was the seven children who were found to have visual issues that were unlikely to be CVI related, meaning the Austin Assessment App also picked up possible ocular visual issues. While this is too small a sample size to draw any conclusions from this finding, it warrants further investigation with a larger number of children who have already diagnosed ocular visual issues. Six children were identified as false positives after being assessed by the Austin Assessment. This can be related to a number of factors including the child being tired, distracted, not understanding what was required during the assessment, and not focusing while completing the assessment. This supports the need for a tool such as the Austin Assessment as a screening tool for CVI related visual issues, with further, more detailed assessment to confirm if a diagnosis of CVI is needed. The investigation into the visual issues found in 15 out of the 23 children in phase two is ongoing.

An important element of phase three was to ensure that the comparison test was similar to the Austin Assessment to ensure that it was assessing the same skills and visual functioning. However, this was challenging, as there is currently nothing similar to the Austin Assessment available with normative range data for this age group. A pen and paper visual search test was deemed the most comparable as it would be able to be completed in a similar manner to the Austin Assessment (both could be completed while the child sat at a table using either a pencil or their finger to complete the assessment) and it didn’t require any additional testing equipment. A number of different pencil and paper tests were considered, including the Sky Search Test, Conjunction Search and Trail Making A and B in addition to the cancellation tests. After evaluation of these tests, it was decided that the cancellation tests were the most comparable to the Austin Assessment as they involved a similar activity of searching for a specific target that was surrounded by other items. While there were several different cancelation test options available (TBCT, SLCT, Star, Bell’s, Six Letter, Color) only the TBCT (5–8 year olds) and SLCT (9–16 year olds) had normative range data available for the age group being assessed [40, 41].

The non-significant kappa result for the 5–8 age group indicates that there was very little agreement between the TBCT and the Austin Assessment. However, this may be due to the fact that the TBCT wasn’t a comparable enough test to the Austin Assessment, as it was developed as a neuropsychological assessment to help diagnose conditions such as unilateral spatial neglect (USN) [40, 41, 53]). While USN is a brain-based impairment defined as the failure to attend or respond to stimuli presented on one side [54], it is only one visual issue under the umbrella of CVI related visual issues. The finding in the phase three research in the younger age group, of 24 children being identified by the Austin Assessment but not the TBCT, therefore suggests that the TBCT was not as sensitive as the Austin Assessment in identifying children with a wider range of CVI related visual issues. This highlights the need for an assessment such as the Austin Assessment as a more sensitive assessment for the full range of CVI related visual issues and not just USN.

For the older age groups (9–12 and 13–18), there was fair to moderate agreement between the Austin Assessment and the SLCT, indicating that the SLCT was an acceptable comparison to the Austin Assessment. While the SLCT test was also developed as a neuropsychological assessment to help diagnose conditions such as unilateral spatial neglect (USN) [40, 41, 53], there was one significant difference between the SLCT and the TBCT. The SLCT had a time element (participants had only 90 seconds to find as many of the six letters as they could), whereas participants completing the TBCT could take as long as they wanted. Recent research from Manley, Bauer [5] has reinforced that children with CVI have slower search and response times, highlighting the need for any assessment or screening for CVI related visual issue to include a timed component. Two of the three variables measured by the Austin Assessment focus on time (overall time taken and dwell time), therefore making it a more sensitive test than the TBCT for CVI related visual issues.

In addition, the finding from phase three that the Austin Assessment has reasonable sensitivity and specificity for each age group with known conditions suggests that the Austin Assessment is an appropriate screening test for CVI related visual issues. However, as already noted in the results section, further investigation on a larger number of children is needed to confirm these preliminarily results. Ideally, this would be conducted with a cohort of children who have already been diagnosed with CVI and more specifically, a dorsal stream dysfunction, and are therefore known to have issues with the higher visual functions or visual perceptual difficulties.

## Conclusion

Although ongoing research is needed to further validate the Austin Assessment, the research reported in this article provides an initial valuation as it indicates that the Austin Assessment App is an effective tool for identifying children who potentially have CVI related visual issues. The purpose of the Austin Assessment is not to definitively diagnose CVI related visual issues in children, it is to detect the children who warrant further assessment to determine if they do have visual issues that impact on their performance when completing the Austin Assessment App. Ironically, the challenge of finding suitable participants for this research has highlighted the need for a tool such as the Austin Assessment. Currently many of the children the Austin Assessment will help to identify are not being recognized and diagnosed, especially the cohort of children with normal visual acuity and issues with their higher visual functions. As with all children with visual issues (ocular or brain based), it is vital that these children do get recognized as having visual issues to ensure that they receive the support they need to be able to succeed in life.
